# Single-site access robot-assisted epicardial mapping with a snake robot: preparation and first clinical experience

**DOI:** 10.1007/s11701-012-0343-6

**Published:** 2012-03-13

**Authors:** Petr Neuzil, Stepan Cerny, Stepan Kralovec, Oleg Svanidze, Jan Bohuslavek, Petr Plasil, Pavel Jehlicka, Frantisek Holy, Jan Petru, Richard Kuenzler, Lucie Sediva

**Affiliations:** 1 Department of Cardiology, Na Homolce Hospital, Roentgenova 2, 150 30 Prague 5, Czech Republic; 2 Department of Cardiac Surgery, Na Homolce Hospital, Roentgenova 2, 150 30 Prague 5, Czech Republic; 3 Medical Device Consulting, Laguna Hills, CA USA; 4 Medrobotics Corporation, Raynham, MA USA

**Keywords:** Ventricular tachycardia, Electroanatomical epicardial mapping, Catheter ablation, Robotic assisted procedure, Flexible robot, Minimally invasive surgery

## Abstract

CardioARM, a highly flexible “snakelike” medical robotic system (Medrobotics, Raynham, MA), has been developed to allow physicians to view, access, and perform complex procedures intrapericardially on the beating heart through a single-access port. Transthoracic epicardial catheter mapping and ablation has emerged as a strategy to treat arrhythmias, particularly ventricular arrhythmias, originating from the epicardial surface. The aim of our investigation was to determine whether the CardioARM could be used to diagnose and treat ventricular tachycardia (VT) of epicardial origin. Animal and clinical studies of the CardioARM flexible robot were performed in hybrid surgical–electrophysiology settings. In a porcine model study, single-port pericardial access, navigation, mapping, and ablation were performed in nine animals. The device was then used in a small, single-center feasibility clinical study. Three patients, all with drug-refractory VT and multiple failed endocardial ablation attempts, underwent epicardial mapping with the flexible robot. In all nine animals, navigation, mapping, and ablation were successful without hemodynamic compromise. In the human study, all three patients demonstrated a favorable safety profile, with no major adverse events through a 30-day follow-up. Two cases achieved technical success, in which an electroanatomic map of the epicardial ventricle surface was created; in the third case, blood obscured visualization. These results, although based on a limited number of experimental animals and patients, show promise and suggest that further clinical investigation on the use of the flexible robot in patients requiring epicardial mapping of VT is warranted.

**Electronic supplementary material:**

The online version of this article (doi:10.1007/s11701-012-0343-6) contains supplementary material, which is available to authorized users.

## Introduction

A “snakelike” robot system has been developed to enable physicians to perform complex procedures intrapericardially on a beating heart without having to open the chest cavity. The CardioARM, developed by Medrobotics (Raynham, MA, USA), allows surgeons to take a curvilinear approach to a surgical target rather than the straight line-of-sight directed approach used in traditional laparoscopic and thoracoscopic surgeries. The multilinked flexible robot contains multiple open device channels to accept a variety of flexible surgical and interventional tools, as well as on-board visualization. The robot needs only a single point of entry and one hand to steer it to the target site under direct visualization. It contains multiple degrees of freedom distributed across its links and is self-supporting, enabling the robot to traverse nonlinear paths in any three-dimensional space without relying on a lumen or organ to maintain its shape [1–6].

The aim of the investigations presented here were to determine whether the CardioARM can be used successfully in the diagnosis and treatment of ventricular tachycardia (VT). For this type of arrhythmia, percutaneous, transvenous, endocardial radiofrequency (RF) ablation has become a secondary treatment strategy when antiarrhythmic drugs and implantable cardioverter-defibrillators (ICDs) fail [7–10]. Yet when critical components of the arrhythmia involve epicardial or subepicardial structures, ablation via the endocardial approach is challenging [10–14]. Prior to the catheter-based endocardial ablation methods, surgical techniques for ablating arrhythmia substrates from an epicardial approach were more common. However, the invasiveness of these techniques, which include open-chest surgery [15, 16], thoracoscopy [17, 18], and entering via epicardial vessels such as the coronary sinus [19], has warranted research into other methods.

Subxiphoid percutaneous epicardial catheter mapping and ablation was first described in 1996, and in the intervening interval this clinical strategy has gained acceptance and is being used at an increasing frequency to diagnose and treat VT when critical components of the arrhythmia may involve structures in the epicardium or subepicardium irregardless if the VT is dependent on the re-entry circuit or focal origin [10–14, 20, 21]. This approach, however, has its own challenges, including gaining access safely and efficiently to the pericardial space without complication, manipulating and controlling catheters originally designed for use within blood vessels, avoiding sensitive structures in which damage can have serious, long-term consequences [22], and delivering enough ablative energy in an environment where electrode contact, presence of epicardial fat, and myocardial scars may present obstacles.

Single-site subxiphoid approaches by both electrophysiologists and cardiac surgeons have previously been described [11, 23–28]. Sosa and Scanavacca [11] described a subxiphoid approach for epicardial mapping; however, their technique (1) requires significant standard fluoroscopically guided catheter manipulation and (2) lacks sufficient visualization of epicardial landmarks. A hybrid surgical approach using the FlexView system avoids both of these issues, but this system can only provide limited access to small regions of the heart [26]. Because of its rigidity, this video-guided approach may be associated with significant hemodynamic compromise and potential arrhythmia, at least in the porcine model [27].

In this report, we present results from both animal and clinical studies of a flexible robot system which we performed in hybrid electrophysiology (EP) surgical settings. The aim of these trials was to demonstrate that the flexible robot can access the pericardial space through a small single-site entry and safely navigate all left and right ventricle surfaces on a beating heart while at the same time enabling direct visualization and mapping of the epicardium with minimal use of fluoroscopy and minimal hemodynamic compromise.

## Materials and methods

### Device description

The CardioARM medical robotic system (Medrobotics, Raynham, MA) is a highly articulated robot platform designed to enable intrapericardial beating heart interventions through a single entry point. Once inserted into the pericardial free space, the flexible robot is designed to access all outer surfaces of the beating heart confined within the pericardium.

**Fig. 1:**
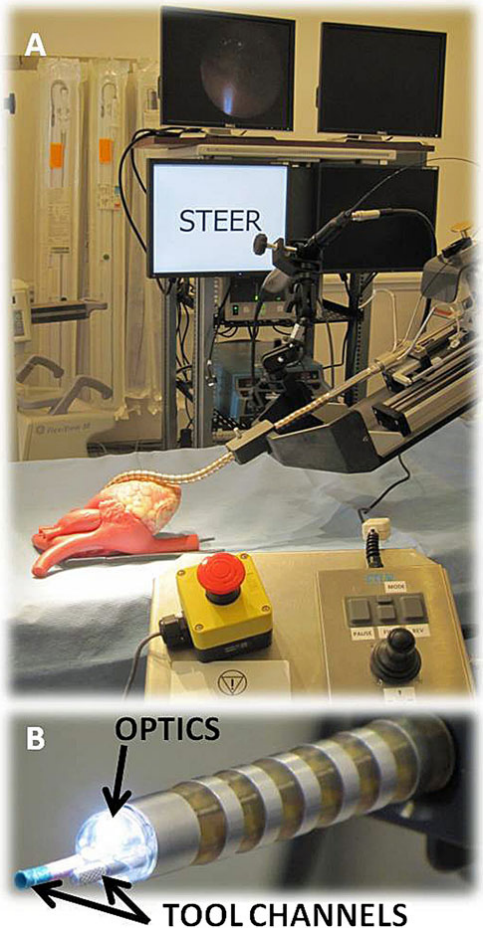
CardioARM medical robotic system. **a** Distal tip of the robotic system showing the “snakelike” device conforming to a surrogate heart model. Screens displaying fiberscope vision, robotic controls, and additional procedural information are in the* background*. The physician control device is shown in* foreground*. **b** Distal tip of the device, which operates in the intrapericardial space. Optics are covered by a dome, and tools channels are shown below

The core technology is the working end of the robot (Fig. 1), which is manipulated through an external control motor and sensor box placed near the patient and is computer-controlled by a physician interface either at the bedside or in a remote application. Previous animal investigational work with an earlier prototype of this device has been described [1–6]. The robot’s flexibility and motion come from having over 30 mechanical linkages in two concentric mechanisms. Each mechanism can be placed into a rigid or a flexible state. By employing a “follow-the-leader” movement strategy [1, 28] with these two alternating states (rigid and flexible), the self-supporting robot can be directed into any shape bounded by the sum of the individual articulating joints and does not require a natural lumen or organ surface to maintain its shape. The robot contains multiple open device channels that can be fitted with a fiberoptic visualization system, a catheter for suction or delivery of additional tools, and a catheter for mapping and ablation (Fig. 1). In these studies a CARTO Thermocool Navistar catheter (Biosense Webster, Diamond Bar, CA) was used to produce bipolar voltage maps of ventricles and deliver radiofrequency energy during ablation therapy. An 8-Fr Softip Guide catheter (Boston Scientific, Natik, MA) was delivered through the remaining channel for suction.

### Animal lab experience

Initial studies of the CardioARM to gain experience with the navigation and ablation tasks were performed on nine female pigs (weight range 40.2–50.0 kg; mean weight 45.2 kg). In the initial study (4 animals), investigators looked at hemodynamic impact and the deliverability of tools without the aid of a mapping system. In the subsequent study (5 animals), full integration of the equipment later to be used in the clinical trial was examined. After each pig had been given general anesthesia, pericardial access was gained through a 3- to 4-cm midline skin incision below the xiphoid process (*n* = 7) or through a small right thoracotomy (*n* = 2). The thoracotomy procedure was attempted to mimic the approach to the human heart (see Discussion section for additional details). The physician removed the xiphoid process and created a 15-mm opening on the pericardium, which was retracted with stay sutures or via Allis clamps. The flexible robot was introduced into the intrapericardial space and guided between the pericardial and epicardial spaces. It was manipulated from the computer-controlled joystick to access various intrapericardial structures, including the ventricular surface and other structures contained within the pericardium. No other incisions were created to allow operation of the robot. Navigation was achieved under fiberoptic camera guidance, which allowed for intrapericardial visualization of anatomical objects and catheters, and was periodically checked by fluoroscopy.

**Fig. 2:**
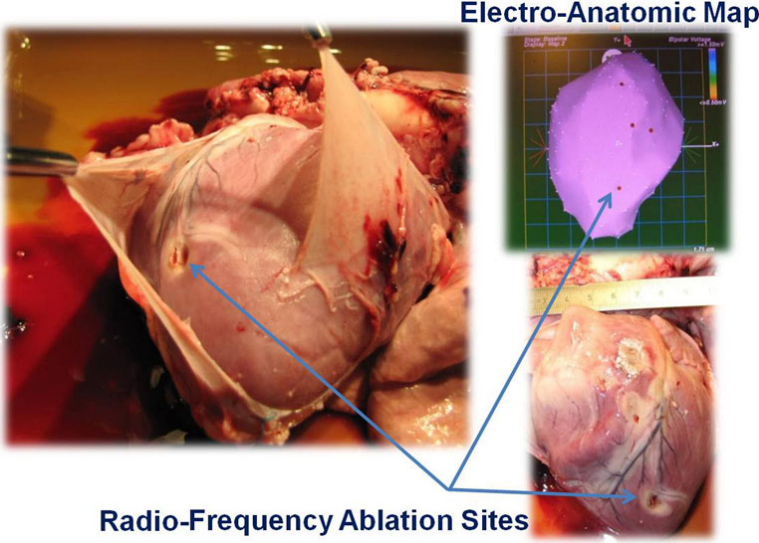
Representative necropsy showing opening of the pericardium, the CARTO map with radiofrequency (*RF*) ablation sites marked during ablation, and corresponding heart following excision

Commercially available RF ablation catheters, which were passed through the robot’s working channels, were used to map and create epicardial lesions. Vital physiological parameters were continuously monitored during the intrapericardial manipulations and ablations. Fluoroscopy was used to verify the real-time position recorded by the CARTO system (Biosense Webster, Diamond Bar, CA), but was not used as a primary mapping method (Fig. 2).

Each animal was euthanized while under general anesthesia and without regaining consciousness. Gross pathology examination of the heart and pericardium was performed after completion of each experiment. All animals received humane care in compliance with the European Convention on Animal Care. The study was approved by the Animal Care and Use Committee.

### Animal results

Mapping equipment was periodically unavailable throughout the animal lab experience. However, investigators performed simulated mapping using a mapping catheter and fluoroscopy. No EP data were collected, but simulated mapping and ablations were performed under vision. These animals (4 pigs) were used to test initial features and examine hemodynamic impact on the heart. Investigators performed fluoroscopy, direct vision, and ablation, and explored anatomical areas. By moving the robot back and forth, they created a simulated map to verify the ability to deliver various ablation tools (usually 7 Fr Thermocool Navistar catheters, but in 2 cases a CryoCath Freezor 7 Fr cryoablation catheter; both Biosense Webster), to visually identify at-risk structures for ablation, to maintain hemodynamic viability of the animal throughout the procedure, and to investigate the heart for signs of damage.

The subsequent studies (5 pigs) involved full system integration, which included fluoroscopy, direct vision, and ablation, and had the additional aim of creating electroanatomical maps using the CARTO system. This study performed not only the functions of the simulated maps in the initial study set, but the results also helped verify the ability to contact electrodes for epicardial electrogram recordings controlled by the CardioARM and to generate a ventricular EP map during sinus rhythm.

Pericardial access was successfully achieved in all animals. The direct visualization guidance feature of the probe allowed successful identification of the anatomical structures and precise catheter delivery to the target sites. A series of isolated ablations was created on the left and right ventricle. RF delivery (*n* = 5 hearts) at an irrigation rate of 2–10 cc/min and within an energy range of 20–40 W was visually observed during application. These ablations were confirmed upon postmortem examination (Fig. 2). No significant changes in vital parameters were detected during probe manipulations, which took 2–3 h in each animal. During manipulation and positioning of the probe, only transient arrhythmias were observed that did not require intervention. In the animal study, we did not attempt to categorize any arrhythmias that did not require an intervention to terminate. Per the protocol, animals were not instrumented to record continuous real time electrocardiograms. All animals remained hemodynamically stable throughout the mapping portion of the procedure. No additional ventricular damage was seen at necropsy other than that from energy intentionally delivered to the tissue during ablation. Fluoroscopy verification was part of the validation of the device and was done in conjunction with the mapping procedure in early animals. In the last few animals, fluoroscopy was delayed until the end of the procedure after full EP maps had been created.

A summary of the animal investigations is presented in Table 1.

**Table 1:** Summary of animal investigations

Porcine ID	Weight (kg)	Surgical approach	Mapping/ablation catheter	Auxiliary catheter	Map attempted/created	Anesthesia time (h:min)
01	40	Subxiphoid	NaviStar^®^ ThermoCool^®^ Catheter 8F	BSX Softp Guider 8F ST	No/NA	4:07
02	44	Subxiphoid	NaviStar^®^ ThermoCool^®^ Catheter 8F	BSX Softp Guider 8F ST	No/NA	2:50
03	50	Subxiphoid	NaviStar^®^ ThermoCool^®^ Catheter 8F	BSX Softp Guider 8F ST	Yes/yes	5:00^a^
04	50	Subxiphoid	NaviStar^®^ ThermoCool^®^ Catheter 8F	BSX Softp Guider 8F ST	Yes/yes	2:15
05	46	Subxiphoid	NaviStar^®^ ThermoCool^®^ Catheter 8F (mapping) Cryocath Freezor 7F, 4 mm (ablation)	BSX Softp Guider 8F ST	Yes/yes	6:45^b^
06	45	Subxiphoid	NaviStar^®^ ThermoCool^®^ Catheter 8F	BSX Softp Guider 8F ST	No/NA	2:13
07	46	Subxiphoid	NaviStar^®^ ThermoCool^®^ Catheter 8F	BSX Softp Guider 8F ST	No/NA	2:30
08	45	Right chest	NaviStar^®^ ThermoCool^®^ Catheter 8F	BSX Softp Guider 8F ST	Yes/yes	3:15
09	41	Right chest	NaviStar^®^ ThermoCool^®^ Catheter 8F (mapping/ablation) Cryocath Freezor 7F, 4 mm (ablation)	BSX Softp Guider 8F ST	Yes/yes	4:00

*NA* not available

^a^Significant intrapericardial adhesion found. Dissection was performed during mapping procedure

^b^Study completed in stages due to PI emergency call and ablation equipment availability

### Human study trial outline enrollment

The single-center, prospective, single-arm study was designed to evaluate the primary safety and feasibility endpoints of peri-procedural major adverse procedural events (MAPE) and technical success, respectively. Patients were followed for 30 days post-index procedure.

Three patients were enrolled in this feasibility trial. All patients were required to have had at least one documented, spontaneous episode of sustained, scar-related VT within the previous 6 months and to be resistant, intolerant, or refractory to at least one Class I or Class III anti-arrhythmia drug. Candidates were considered ineligible for any of the following reasons: previous cardiac surgery or documented pericarditis; large body habitus or morbid obesity that would preclude obtaining subxiphoid access to the heart; other illness that might cause the patient to be noncompliant, confound data interpretation, or be likely to live less than 6 months after enrollment. Prior to placing the flexible robot in the patient, the pericardial space was explored to rule out adhesions.

The primary safety endpoint for this study was peri-procedural MAPE, defined as death, cardiac perforation, coronary artery occlusion (due to energy delivery), or cardiac tamponade. Subjects were to be evaluated at 48 h post-procedure or at the time of hospital discharge, whichever occurred first. The secondary safety endpoints were: (1) MAPE evaluated 30 days post-procedure and (2) overall adverse events, whether serious or nonserious. The primary feasibility endpoint was technical success: the ability of the CardioARM to navigate the EP catheters and create a CARTO electroanatomic map of the epicardial surface of the ventricles.

This study was conducted in accordance with the rules of the institution’s ethics committee, country-specific regulations, and the Helsinki Declaration of 1975, as revised in 2008.

### Clinical procedure

Each patient underwent the procedure in a hybrid EP-surgical suite under general anesthesia. After induction of general anesthesia, the intended subxiphoid and femoral access sites were prepped and draped using sterile technique. Percutaneous femoral arterial/venous sheaths were inserted, and EP multielectrode catheters were advanced into the right atrium, coronary sinus, and right ventricle apex. The surgeon then made a 3- to 6-cm subxiphoid incision and performed minimal tissue dissection down to the pericardial level. A 15-mm pericardiotomy was performed under direct visualization over the right ventricle and suspended with retaining sutures (Fig. 4c).

**Fig. 3:**
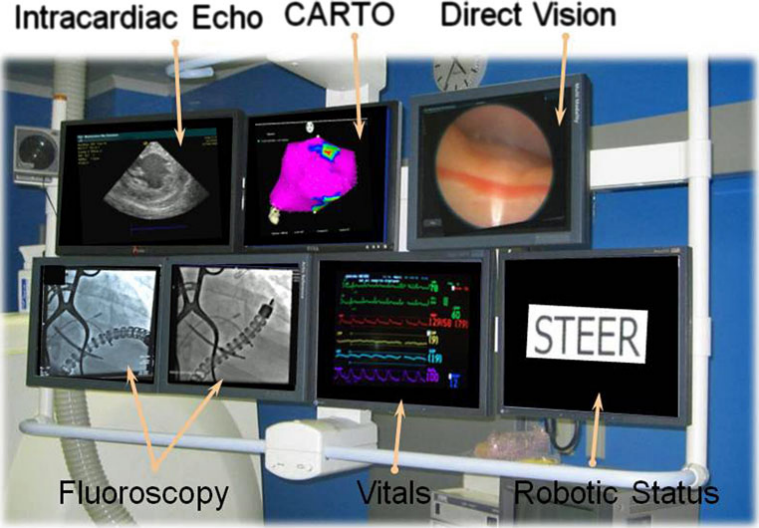
Monitors used during the clinical trial procedure. Information displayed include intracardiac echocardiogram, CARTO, fiberscope vision, fluoroscopy (2 monitors), vitals, and robotic status information. Images shown have been superimposed on the monitor bank

The flexible robot was inserted into the pericardial space and navigated under fiberoptic camera guidance while its position was being periodically validated with fluoroscopy. A Thermocool Navistar catheter was passed through one of the robot’s internal working channels and was used to create ventricular electroanatomical maps using the CARTO mapping system. Vital physiologic parameters and an intracardiac echocardiogram were continuously monitored throughout the procedure. Electroanatomical cardiac data were collected from a catheter deployed through the flexible robot, direct vision was seen from the distal tip of the device, and periodic fluoroscopic images were taken at the discretion of the investigator (Fig. 3).

Delivery of ablative therapy through the investigational device was left to the discretion of the investigator. After the procedure, each patient had a short 9 Fr pigtail catheter (NGC Medical, Como, Italy) placed intrapericardially through the original incision to help drain any fluid that might accumulate.

### Human results

The first patient enrolled was a 43-year-old Caucasian female (weight 82 kg, height 182 cm) in New York Heart Association (NYHA) Class I heart failure, with an ICD and a history of multiple unsuccessful attempts of endocardial RF ablation. The second patient, a 70-year-old Caucasian male (weight 96 kg, height 178 cm) in NYHA Class II heart failure, also had an ICD. His history included unsuccessful attempts at RF ablation for both atrial fibrillation and VT, and percutaneous coronary intervention and cardioversion. The third patient was a 67-year-old Caucasian male (weight 86 kg, height 180 cm) in NYHA Class II heart failure, with a left ventricular ejection function of 25% and an occurrence of VT 17 days prior to enrollment. His history included one myocardial infarction, one transient ischemic attack, and ischemic heart disease. The source of the VT in the latter two patients was suspected to be epicardial.

All three subjects, after subxiphoid pericardial access was obtained, underwent CARTO electroanatomic mapping of the epicardial surface of the right and left ventricles, using the CardioARM fitted with a Cordis Biosense Webster Navistar Thermocool catheter.

Total procedure time, CARTO mapping time, and total fluoroscopy time ranged from 96 to 219 min, from 50 to 159 min, and from 11 to 17 min, respectively.

Technical success was achieved in the first and third patients, but not in the second, where visualization was obscured by blood originating from outside the pericardial space. The device was removed to inspect and conventionally control bleeding. Once under control, no attempt was made to reintroduce the investigational device. Catheter mapping and ablation was performed using a standard manual technique. No patients experienced a peri-procedural MAPE, as defined in the safety endpoint, which was subsequently confirmed at the 1-week and 30-day follow-ups. In all trials, a total of three nonserious adverse events occurred between the time of enrollment and the 30-day follow-up, but none were attributed to the investigational device.

A summary of clinical work is presented in Table 2.

**Table 2:** Summary of clinical work

Patient ID	Age (years)	Height (cm)	Weight (kg)	Body mass index	NYHA class	Procedure time (h:min)	Robotic carto time (h:min)	Ablation performed	Fluoroscopy time (min:s)
01	44	182	82	24.8	I	3:39	2:39	None performed	11:13
02	71	178	96	30.3	I	1:41	NA^a^	Conventional	13:00
03	68	180	86	26.5	II	1:36	0:59	Conventional	17:00

*NYHA* New York Heart Association

^a^Mapping/ablation completed conventionally due to extrapericardial bleeding

## Discussion

### Animals

Single-site pericardial access, navigation, mapping, and ablation were successful in all animals, without hemodynamic compromise. Anatomical structures could be identified in all animals, and fluid management within the pericardium was successfully managed to maintain direct view of the space during the entire procedure. Electrode contact and EP maps were comparable to those of other epicardial approaches previously performed within this institution, and various imaging modalities were integrated with the CardioARM without difficulty. All procedures could be performed using only the robot’s own direct visual guidance, with or without fluoroscopic assistance. Postmortem evaluation was consistent with previous work in manual percutaneous epicardial ablation [9].

### Human study

The aim of this clinical investigation, the first known human use of a snake robot for right and left epicardial ventricular mapping, was to evaluate whether the consistent positive findings from the porcine model were translatable to the human anatomy. The intent of this trial was to simply demonstrate initial feasibility of entering the pericardial space from a single small access point and, in that space, of safely navigating the robot around a beating heart. Comparisons to conventional procedures were not examined in this study.

**Fig. 4:**
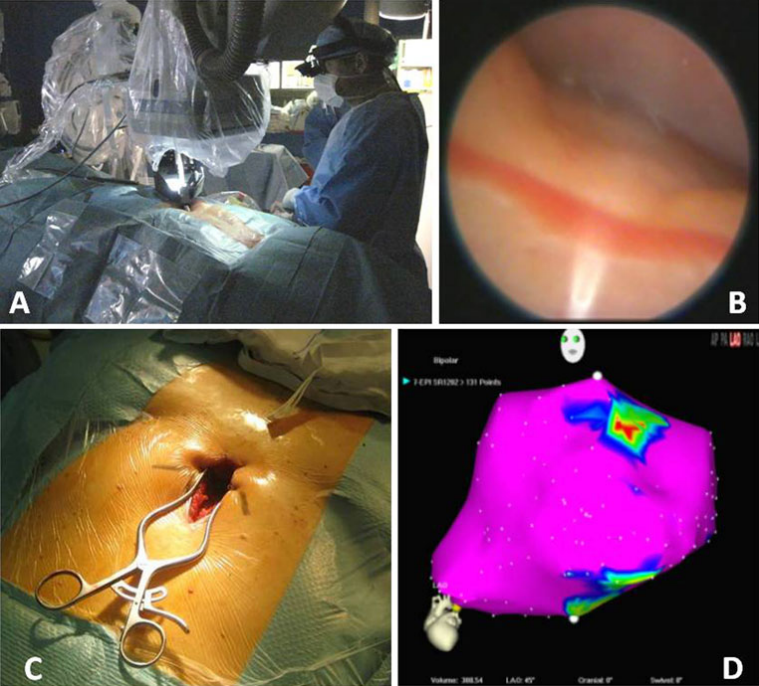
Select images during the clinical enrollment. **a** External view of the setup, **b** intrapericardial view, **c** subxiphoid access view, **d** electroanatomical CARTO map at the completion of the procedure

Investigators were able to access the pericardial space through a subxiphoid entry and navigate the epicardial surface of the ventricles without inducing clinically significant hemodynamic or arrhythmogenic effects. The robot provided direct images of a standard EP mapping catheter, a suction catheter, and the cardiac surface anatomy of a beating heart. In two of three subjects, EP maps were successfully created with the assistance of the CardioARM (Fig. 4). The robot exhibited a favorable safety profile, with no adverse events attributed to the device. However, based on the small sample size, it is impossible to draw any conclusions other than the initial feasibility.

In the first patient, the robot was challenged when directed to map at the apex of the heart. However, to improve the setup, it was believed that the robot could be better directed towards the apex, or physicians could consider an alternative surgical approach, such as the left chest to better reach around the apex.

Technical success was not achieved in the second patient. After a sparse map had been started early in the procedure, a small amount of blood obscured visualization. Following device removal, the source was discovered outside the pericardial space, likely generated during the minimally invasive subxiphoid preparation. Once the bleeding was controlled, mapping was concluded manually by using fluoroscopy. Only a minimal amount (not recorded) of blood was suctioned intraoperatively, and the intrapericardial pigtail place at the conclusion of the procedure was removed in <24 h with no unusual amount of fluid collected.

### Limitations and future considerations

The porcine studies using subxiphoid access were a good model to prepare surgeons for examining CardioARM use in humans, and they provided insight into the learning curve. Because the different orientation of the porcine heart limited analogous understanding of the device in humans, investigators also tried right chest access in two cases, but the full range of this approach was not explored. The animal studies were a reasonable investigation model, but they did not present all of the challenges expected in a clinical setting. Finally, histology was never performed. These factors suggest possible avenues for further study.

Because the clinical trial was so small, certain limitations were inevitable. The three patients fell within a fairly narrow range of body type, which may have facilitated navigation of the entire epicardial surface in all cases. VT ablations were permitted according to the protocol, but they were never performed, and the procedure was limited to mapping only. It is untested how the investigational device would perform during an ablation if a hemodynamic instability should occur following the delivery of ablative energy. The failure to reach technical success in the second patient underscored the importance of maintaining a pristine field when visualizing in a confined space around a beating heart. Further investigation would clarify these issues.

Several potential technical advances would increase the range of the flexible robot’s applicability. It is possible that in patients with NYHA Class IV heart failure, their enlarged hearts would create tighter spaces and theoretically lead to hemodynamic problems. Reducing the size of the device from its current diameter of 12 mm would increase the feasibility of treating such patients. The device maintains its shape during navigation, but also has a safety feature (not used in this investigation) that enables it to go limp thereby ensuring that it can be quickly and safely removed should the need arise, such as in hemodynamic instability. However, this mechanism, in the device’s current form, limits the operator’s ability to pull it back and pick up electroanatomical points quickly. Therefore, merging the safety feature with enhanced efficiency is a possible improvement for the device. Finally, developing a percutaneous approach could leverage the flexible robot to a diverse set of users and minimize the likelihood of surgical complications. Instead of relying on surgical access, as in this study, a specific access kit targeted to electrophysiologists, interventionalists, and cardiac surgeons could increase the practicality of the product across all specialties.

## Conclusion

Following the encouraging results of the porcine studies, the small clinical trial demonstrated the potential feasibility and safety of using a flexible robot system for minimally invasive epicardial mapping in patients with previous multiple failed attempts using an endocardial approach. Both clinical investigation and improved percutaneous access are required to demonstrate efficacy.

These results, while limited, show strong promise and suggest that further clinical investigation of the CardioARM in patients requiring epicardial mapping of VT is warranted.

## Electronic supplementary material

Below is the link to the electronic supplementary material.

Supplementary material 1 (QT 5194 kb)
